# The oral vaccination with *Paenibacillus larvae* bacterin can decrease susceptibility to American Foulbrood infection in honey bees—A safety and efficacy study

**DOI:** 10.3389/fvets.2022.946237

**Published:** 2022-10-17

**Authors:** Franziska Dickel, Nick Maria Peter Bos, Huw Hughes, Raquel Martín-Hernández, Mariano Higes, Annette Kleiser, Dalial Freitak

**Affiliations:** ^1^Institute of Biology, Karl-Franzens University, Graz, Austria; ^2^Dalan Animal Health, Inc., Ojai, CA, United States; ^3^Independent Researcher, Taastrup, Denmark; ^4^Echo Veterinary Consulting, Saint-Hippolyte, QC, Canada; ^5^Laboratorio de Patología Apícola, Centro de Investigación Apícola y Agroambiental (CIAPA), Instituto Regional de Investigación y Desarrollo Agroalimentario y Forestal (IRIAF), Consejería de Agricultura de la Junta de Comunidades de Castilla-La Mancha, Marchamalo, Spain

**Keywords:** vaccination, honey bees (*A. mellifera*), *Paenibacillus larvae*, trans-generational immune priming, clinical trial

## Abstract

Pollination services to increase crop production are becoming more and more important, as we are facing both climate change and a growing world population. Both are predicted to impact food security worldwide. High-density, commercial beekeeping has become a key link in the food supply chain, and diseases have become a central issue in hive losses around the world. American Foulbrood (AFB) disease is a highly contagious bacterial brood disease in honey bees (*Apis mellifera*), leading to hive losses worldwide. The causative agent is the Gram+ bacterium *Paenibacillus larvae*, which is able to infect honey bee larvae during the first 3 days of their lives. It can be found in hives around the world with viable spores for decades. Antibiotics are largely ineffective in treating the disease as they are only efficient against the vegetative state. Once a hive shows the clinical manifestation of the disease, the only effective way to eradicate it and prevent the spread of the disease is by burning the hive, the equipment, and the colony. Because of its virulent nature and detrimental effects on honey bee colonies, AFB is classified as a notifiable disease worldwide. Effective, safe, and sustainable methods are needed to ensure the wellbeing of honey bee colonies. Even though insects lack antibodies, which are the main requisites for trans-generational immune priming (TGIP), they can prime their offspring against persisting pathogens. Here, we demonstrate an increased survival of infected honey bee larvae after their queen was vaccinated, compared to offspring of control queens (placebo vaccinated). These results indicate that TGIP in insects can be used to majorly enhance colony health, protect commercial pollinators from deadly diseases, and reduce high financial and material losses to beekeepers.

**Classification:**

biological sciences, applied biological sciences

## Introduction

Sustainable solutions to maintain and increase crop yields globally, in the face of climate change and the intent to lower the carbon footprint, are essential to address food security for a growing population. Commercial beekeeping is an integral part of these efforts and honey bee pollination is a vital component of our food supply chain (1–4). Managed honey bees (mostly *Apis mellifera*) are essential for the pollination of various plants, which makes them valuable for functioning ecosystems, and productive livestock of increasing importance (5, 6). The majority of high-nutrient-density crops are pollination-dependent. Some crops, like almonds, are 90–100% dependent on honey bee pollination (6, 7). Furthermore, increasing concern around the losses of wild pollinators heightens the need for reliable pollination services (8, 9). While honey bees are the most economically valuable pollinators, they are threatened by a variety of pathogens (5). Yet, safe and effective prophylactic solutions for disease prevention are lacking. The development of new disease control measures for commercial beekeeping needs to be a critical part of sustainable agriculture and livestock management.

One of the most detrimental diseases worldwide is American Foulbrood (AFB). It is caused by the gram-positive spore-forming bacterium *Paenibacillus larvae*. The spores can last for decades in the environment, staying virulent the entire duration, and thus posing a continuous threat to honey bee colonies (10, 11). Honey bee larvae are susceptible to infection with AFB during the first 24–72 h after hatching (12). Spores from diseased broods are picked up by worker bees, which carry and spread the infectious spores throughout the hive. In addition, spores are carried outside the hives by forager bees and can spread between hives during robbing, which occurs when weakened and diseased hives are robbed for their honey by bees from other hives (11, 13).

AFB disease management is currently limited to the burning of the diseased hive and colonies, or in some countries, prophylactic feeding of antibiotics to the hives (5, 14). With the growing concern of antibacterial resistance and antibiotic contamination of food and feed, the use of antibiotics in livestock, including honey bees, is under growing scrutiny around the world. In most countries, such as the USA, prophylactic use of antibiotics in livestock is not permitted and use is restricted to acute or at-risk cases (15). In contrast, European Union has a zero-tolerance policy for antibiotic use in honey bee management (16, 17). There is an urgent need for a sustainable, non-chemical solution to prevent diseases and curtail colony losses. Vaccines, based on inactivated bacteria, have been proven to be an effective method for disease prevention in many livestock species, as well as poultry and aquaculture, and can be developed across all the classes of pathogens, i.e., bacteria, parasites, viruses, and fungi (18).

While the insect's immune system is lacking antibodies, it has been shown that they can prime themselves (immune priming) and show remarkable resistance against diseases they have previously encountered (19, 20). One of the fascinating and long-debated aspects of the insect immune system is the ability to transfer the knowledge of pathogen encounters from one generation to the next. The phenomenon has been coined *trans-generational immune priming* (TGIP) (21, 22). The immune priming resembles adaptive immune mechanisms in vertebrates, though lacks the specific immune mechanisms that are mediated by antibodies, cytotoxic T lymphocytes, and other epitope-specific immune mechanisms (18). The mechanism behind this antibody-free immunological priming has been a mystery for a long time. The work done in honey bees established that information about the disease agent can be transferred to the next generation with the help of the egg yolk protein Vitellogenin carrying immune elicitors, such as pieces of bacteria (23, 24). Transfer of immune elicitors might not be the only way how insects are preparing their offspring to fight off diseases. The transfer of other signals, such as mRNA and proteins as well as epigenetic factors, has also been proposed (21). The advances in the understanding of immune priming mechanisms have enabled the field of insect health to start developing preventative methods for disease management. This is particularly relevant for managed pollinators, who are kept at high densities and are subjected to nomadic animal husbandry where the risk of the spread of diseases is significantly increased (25).

It has previously been shown that honey bee larvae can be protected against homogeneous *P. larvae* infections when the queens were injected a heat-killed *P. larvae* bacteria of the same strain (26). However, this method has practical limitations and can be harmful to the queen bee as the injury and stress will have a considerable effect on the health and survival of the queens. Oral infection is the natural way of infection for most bacterial pathogens, and thus was used by Ory et al., to test a possible TGIP effect in honey bees against *Melissococcus plutonius* (causative agent of European foulbrood). The team used live, unattenuated *Melissococcus plutonius* delivered in a liquid sugar solution as a single dose directly to the queen. In this case, the authors could not show evidence for TGIP against *M. plutonius* (27). While this approach mimics the natural environment in hives and colonies where the queen is exposed to live pathogens, the approach still holds limitations for a broad adaption as the exposure of colonies to virulent pathogens holds a higher risk for accidental infection. A more economically feasible method of priming insects with the potential for broader industry adaptation would be an oral delivery of the killed pathogen. In addition, dosing is of the essence in the case of vaccines, as a too-low dose might not provide any immunity against the disease. To show the potential for broader protection of a vaccine product, it is important to expose animals to disease-causing pathogens that differ from the pathogen strain in a heterologous challenge set-up. In this study, we report a very first safety and efficacy vaccine trial in honey bees using a heterologous challenge and demonstrate that oral administration of an inactivated AFB bacterin to the queen bees is safe and induces protection in the next generation larvae. This method provides the basis for a first-ever insect vaccine to enhance colony health, which is essential to decrease the economic impact of diseases on beekeeping operations, increase the health status of apiaries, and reduce diseases' spillover to wild pollinators.

## Materials and methods

### Study sites, animals, and the lab efficacy trials

Two separate trials were carried out in two different locations. Study site A was in Graz Austria, with 20 AFB-bacterin vaccinated colonies and 10 Placebo hives established in the university beeyard. Study site B was in Marchamalo Spain, with 15 AFB-bacterin and 15 Placebo hives established in the IRIAF beeyard. In Austria, honey bee subspecies *Apis mellifera carnica* was used and in Spain *Apis mellifera iberiensis*. Larval samplings to estimate the laboratory efficacy of the vaccine to prevent the infection were carried out twice in both locations, in both cases only hives having enough larvae (30 per hive) were enrolled in the study (the rest were excluded as the queen failed to produce the brood at the relevant time point).

### Bacterin preparation and vaccination

The bacterin preparation was derived from a *P. larvae* strain (original ID 9815), originally isolated in 2018 and obtained from the Bee Diagnostic and Diseases Laboratory, USDA—Agricultural Research Service, Beltsville Agricultural Research Center-East, MD. The bacterin was a proprietary aqueous suspension of inactivated *P. larvae* vegetative stage bacilli provided by Dalan Animal Health. The bacterin has passed all regulatory testing of purity and was tested and found to be of ERIC I genotype. Bacteria were enumerated by flow cytometry and OD_600_ before inactivation. The bacterin was blended with queen feed (48 ml corn syrup per 500 g powdered sugar) at a ratio of 1 ml per 100 g (or control using 1 ml of water per 100 g queen feed). The queens were received from local queen breeders already caged in queen cages with each 6–10 attendees at both study sites, probably closely related but not sister queens. Queens in both locations were vaccinated (Location A: AFB-bacterin *n* = 32, Placebo *n* = 16: Location B: AFB-bacterin *n* = 15, and Placebo *n* = 15) for 8 days by feeding them 6 g of the queen feed in queen cages in the laboratory (darkness and room temperature). From these, 20 vaccinated queens and 10 placebo queens were selected (by the person who carried out the blinding process, see below) and released into five-frame nucleus hives in Location A and all 30 queens were released into hives in Location B. The experiment was conducted using a double-blinded design. The queen feed was divided into two different treatments (bacterin and placebo) and randomly labeled from 1 to 48 in location A and 1 to 30 in location B by a specifically assigned person, who was not participating in the study. The researchers carrying out the experiments in both locations were blind to the treatments given to queens.

### Challenge material preparation and challenge of larvae

After at least 18 days post hive placement, frames with 0–36 h old brood were brought to the laboratory for challenge testing. Larvae were grafted onto petri dishes prepared with droplets of larvae food (50% royal jelly, 6% glucose, 6% fructose, 1% yeast extract, and 37% water). The larvae food was also used to prepare challenge material by diluting spores of a heterologous *P. larvae* strain (ID 10159, first isolated by BDDL) to a final concentration of 10,000 spores/379 μl food droplet. Spores were propagated on MYP+glucose agar (described above), harvested *via* washing 3× with 5 ml ice-cold autoclaved H_2_O, washed by three centrifugation steps, and the pellet was then diluted in autoclaved H_2_O. Spores were counted using a hemocytometer (Buerker, Marienfeld) and diluted to a spore stock concentration of 308,000,000 spores per μl.

Subsequently, one set of larvae (≥30 individuals per hive) was challenged with spore solution, and one set without the spores to assess background mortality. In addition, vigorous testing using the environmental controls (larvae from placebo hives and control treatment) was done at all times as quality control to obtain data. In the case, where higher than 80% mortality in environmental controls was recorded, the data were excluded from further analysis due to mortality *via* handling. Five larvae per food droplet (15 larvae per Petri dish) were used, whereas three droplets of 379 μl were placed on one petri dish. Larvae were monitored on a daily basis for 8 days following the challenge, placing them on fresh food droplets (without spores) every second day (Day 2 = 700 μl, Day 4 = 800 μl, Day 6 = 1,000 μl, and Day 7 = 1,000 μl). Petri dishes were kept in dark at 37°C and ~60% humidity. Dead larvae were removed, and data were recorded in data capture forms.

### Statistical analysis

All analyses were conducted in R-Studio [version 1.2.1335; (28, 37)]. Two separate data analyses were conducted on each dataset. To establish the efficacy of the bacterin, prevented fractions were calculated using the RRsc function [PF package; (29)]. Generalized linear models with binomial distributions, combined with *post-hoc* contrasts were used to estimate a *p*-value for the effect of the bacterin. The general linear model contained the number of dead and alive larvae per hive [cbind (dead, alive)] as the dependent variable, and hive treatment (bacterin and placebo) and larvae treatment (control and challenge) as independent variables. *Post-hoc* comparisons, including adjustments for False Discovery Rate (FDR), were conducted using the emmeans package [emmeans package; (30)].

## Results

### Safety evaluation

During the observation period in the laboratory for over 8 days, all queens survived and exhibited no signs of stress (wing beating, movement, feeding, and survival) to ingestion of the vaccine or placebo (Table 1). Once the honey bee queens were placed into hives, there was an expected hive loss due to reasons, such as environmental conditions and the frequent opening of hives to retrieve larvae (monitoring period = 3 months). There was no statistically significant difference between vaccinated and control hives (Table 1).

**Table 1:** The survival of the queens during vaccination.

**Treatment**	**Location**	**Queens**			**Hives**		
		**No**.	**Alive**	**Dead**	**No**.	**No. Dead**	**% Loss**
AFB-bacterin	Site A – Graz, Austria	32	32	0	20	5	25
	Site B – Marchamalo, Spain	15	15	0	15	4	26.7
Placebo	Site A – Graz, Austria	16	16	0	10	3	30
	Site B – Marchamalo, Spain	15	15	0	15	5	33.3

Effect of Queen Vaccination (Treatment) on Queen survival during 8-day vaccination period, and hive survival after queens placement to the hives at study site A—Graz, Austria and study site B—Marchamalo, Spain.

### Efficacy

#### Study site A

**Figure 1:**
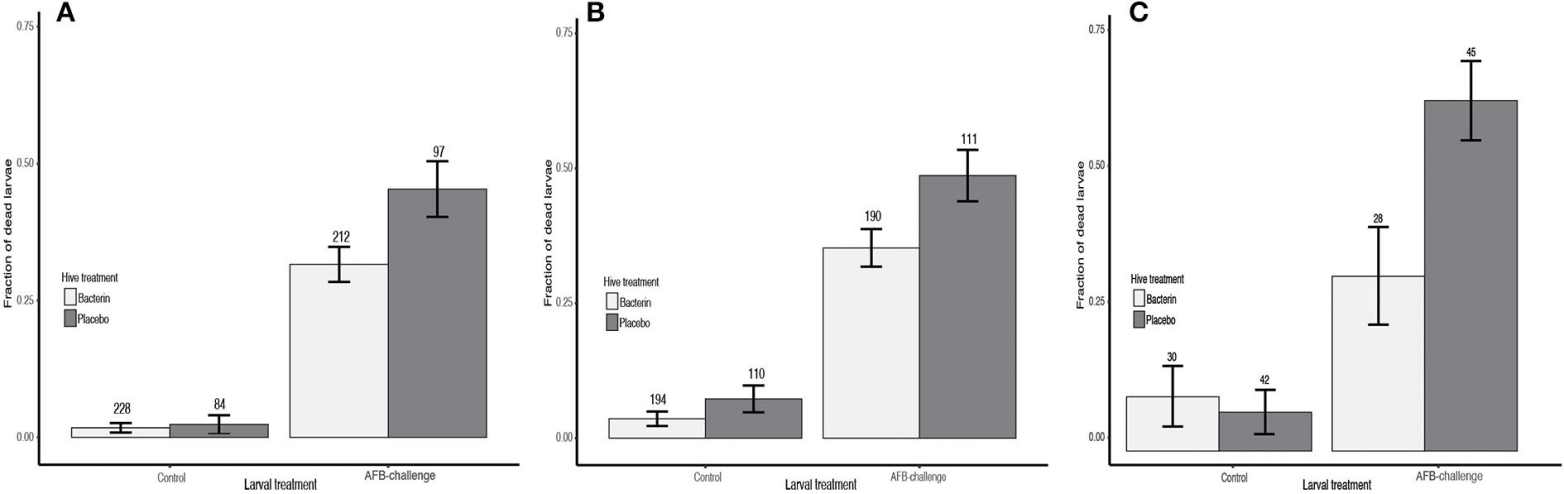
The efficacy of the vaccination to prevent the infection with AFB. Prevented Fraction showing the vaccination efficacy of *P. larvae* bacterin (light grey bars) vs. Placebo (dark grey bars) in a heterologous challenge, as mortality of larvae after 8 days. **(A)** Study Site A—first challenge, **(B)** Study Site A—second challenge, and **(C)** Study Site B—second challenge.

The hives were challenged twice. In the first challenge, seven placebo hives and 16 AFB-bacterin-vaccinated colonies were enrolled in the study (seven hives had queen failure). Oral vaccination with AFB-bacterin prevented 30% of deaths in larvae after heterologous challenge for 8 days, compared to placebo treatment (Table 2; PF = 0.303). The AFB-bacterin vaccination significantly decreases the mortality of larvae when challenged with a heterologous challenge, *p*-value = 0.02; compared to larvae from a Placebo control hive (Figure 1A, Table 2). A control challenge showed that the oral AFB-bacterin vaccination has no negative effect on the survival of larvae (Figure 1A and Table 2; *p* = 0.72). The second round of challenge (19 days after the first challenge) resulted in similar results, preventing 28% of death in larvae (Table 2; PF = 0.275), with seven placebo hives and 13 AFB-vaccinated colonies (10 hives had queen failure). Mortality was significantly decreased in AFB-challenged larvae from AFB-bacterin vaccinated colonies, compared to placebo control hives (Table 2; *p* = 0.02). The AFB-bacterin vaccination shows no negative effect on the general survival of the larvae (environmental controls) (Figure 1B and Table 2; *p* = 0.16).

**Table 2:** The efficacy of the vaccination.

**Contrast**	**Challenge treatment**	**Challenge No**.	**Odds ratio**	**SE**	***p*** **-value**	**PF**
**Site A – Graz Austria**						
AFB bacterin vs. Placebo	*P. larvae*	1	0.56	0.14	0.02	0.303
		2	0.57	0.14	0.02	0.275
	Control	1	0.73	0.64	0.72	-
		2	0.48	0.25	0.16	-
**Site B – Marchamalo, Spain**						
AFB bacterin vs. Placebo	*P. larvae*	1	0.26	0.13	0.01	0.501
	Control	1	1.44	1.23	0.67	-

General linear model with *post-hoc* contrast at both study sites (Site A—Graz, Austria, Site B—Marchamalo, Spain) and all challenges conducted (two challenges at Site A and one challenge at Site B). The contrast shows the queen treatments which are compared to each other (bacterin = AFB vaccination, Placebo = control vaccination), and Challenge treatment shows whether larvae were treated with the heterologous challenge (*P. larvae*) or placebo (Control). A p-value of 0.05 or less means a significant difference in mortality (SE = standard error).

#### Study site B

The third challenge was carried out in study location B. The challenge experiment used three placebo and two AFB-bacterin vaccinated colonies (the rest of the hives had queen failure). In this study, oral vaccination with AFB-bacterin prevented 50% of deaths in larvae after heterologous challenge for 8 days, compared to placebo treatment (Table 2; PF= 0.501). The AFB-bacterin vaccination shows no negative effect on the general survival of the larvae (environmental controls) (Figure 1C and Table 2; *p* = 0.01).

## Discussion

To our knowledge, we report here a first-ever successful randomized placebo-controlled double-blinded trial using a classical vaccination approach to protect honey bees against a disease. In our study, we administered a killed bacterin orally to honey bee queens and observed up to a 50% increase in disease resistance in their offspring in the laboratory. Our bacterin can be classified as a breeder vaccine, in which case the vaccination is carried out *via* parental animals (31). To our knowledge, this was the very first insect vaccine trial and constitutes a turning point in disease management in insects.

Vaccination of the bee colonies appears to be a safe way to prevent the probability of the larvae to succumb to the disease. In addition to significant lab efficacy in preventing the disease, we saw no difference in hive losses between placebo and bacterin-treated colonies. Somewhat higher in-season hive loss (about 30%) could have been caused by frequent opening and removal of the brood from the hives, which is no doubt a very stressful event for the bees.

The current study followed a trial design commonly used in animal vaccine efficacy trials to demonstrate lab safety and efficacy using a heterologous challenge strain. Due to the highly contagious nature of AFB, hives cannot be challenged in the wild. Nor can bees (which are wild animals) be kept in captivity, as a single hive contains between 10,000 and 30,000 (in some cases up to 80,000) individual bees, who would need to fly out and forage in a radius of up to 10 km (32, 33). It is important to note that when using social insects, the experimental unit is the hive, rather than an individual bee. This creates a number of constraints on the sample size and the number of hives that can be enrolled in the study. In the interests of reducing the environmentally induced variation in the experiment, we aim to keep the conditions of the trial as similar as possible. With wild animals, such as bees, we can typically house no more than 40 hives in one location. In addition, other random factors need to be considered, such as differences in where individual bees fly to forage, random visits from other hive yards, pesticide exposure, or attacks by natural enemies (e.g., wasps and hornets).

To counteract the limited sample size and natural variation, we sample many larvae at the same time point, and we carry out multiple lab efficacy trials. The hive is still the experimental unit in which the individual larvae are removed at an early age (≤ 1 day) and exposed to the disease to mimic the natural disease condition. Throughout the study, honey bee colonies are exposed to environmental conditions which may influence the availability of larvae at any given time (see above for details). Furthermore, genetic differences between the hive, partially explained by free mating practice, where queens are allowed to mate freely during the nuptial flight, can affect brood availability. In addition, the placement of hives in the yard can result in variations in the number of right-age larvae between hives at any given time. Larvae from multiple days cannot be included in one experiment and only larvae on a given day can be included. The reason behind this is the differences in the weather, food, and pesticide exposure between different time points induce variables that can skew the results. Therefore, to ensure the availability of sufficient larvae of the right age on a particular challenge day is always with a certain risk of not having the possibility to enroll all the hives in the trial at any time. We use the term queen failure to describe the lack of right-aged larvae on a given sampling day, though she might have had enough brood at some other time. Also due to the double-blind trial design, the experimenters collecting the larvae never know which treatment the hives received, and unavoidably this can lead to unbalanced placebo vs. bacterin treatment groups in the final data sets.

The data presented here indicate that infection with AFB can be decreased by about 30–50% in the laboratory conditions after vaccination of the queens. Interestingly, it has been shown that virulent spores are present even in asymptomatic hives, having an average of 158 spores per bee; however, an increase in spore loads of 30% (to about 228 spores per bee) can lead to a clinical outbreak of the AFB (11). Erban et al. and Peters et al. also found in their studies that hives without clinical symptoms can have AFB spores up to a certain concentration. However, clinical symptoms will manifest, and hives need to be eliminated when the spore level is increased by about 30% or more (34, 35). All this suggests that even a modest decrease in the infection level will keep the disease from manifesting in the hives. It has been shown by using molecular diagnostic methods that AFB spores are also present in asymptomatic hives (36), which could break out into a clinical manifestation at any moment. From this, we can predict that vaccines and bacterins with even relatively moderate efficacy will have the potential to become an essential management method in honey bees to prevent diseases in the wild. It is important to note that in this study, the larvae were exposed to a heterologous challenge rather than using spores from the vaccine strain as a challenge. Particularly, in migratory beekeeping operations where hives are constantly exposed to different stressors and disease strains a vaccine that has the potential for broad protection is essential. As immunity in insects does not rely on the amplification of responses through the epitope-specific clonal expansion of immune-reactive cells or antibodies, efficacy levels may not always reach the same levels as those in vertebrate studies when faced with a large challenge of pathogenic bacilli. Despite this, our data indicate that the innate immune response in insects is sufficient to decrease spore count to a level that there is a significant effect on clinical disease and AFB epidemiology. Using two different locations and two different subspecies of honey bees, we demonstrate for the first time that insect vaccination can constitute an effective prophylactic method for the management of invertebrate diseases. Large-scale longitudinal field efficacy trials will be required to determine the overall effects of oral honey bee vaccination on colony health.

## Data availability statement

The raw data supporting the conclusions of this article will be made available by the authors upon request.

## Ethics statement

Ethical review and approval was not required for the animal study because study was conducted using an invertebrate model - honey bee (*Apis mellifera*). Experiments with invertebrates are not regulated by law.

## Author contributions

FD, AK, HH, and DF designed research and wrote the article. FD, RM-H, MH, and DF performed research. NB analyzed data. All authors contributed to the article and approved the submitted version.

## Funding

The study was sponsored by Dalan Animal Health, Inc.

## Conflict of interest

The authors declare that the research was conducted with financial support of Dalan Animal Health, Inc. Author HH was employed by Echo Veterinary Consulting. Author NB was consultant to the study. Authors FD, AK, and DF are founders of the sponsor company. Authors FD and DF were involved in sample collection, and analyses of data. All authors were involved in the design of the study and in the decision to publish the results as indicated in the Author Contributions.

## Publisher's note

All claims expressed in this article are solely those of the authors and do not necessarily represent those of their affiliated organizations, or those of the publisher, the editors and the reviewers. Any product that may be evaluated in this article, or claim that may be made by its manufacturer, is not guaranteed or endorsed by the publisher.
